# Interplay Between Sickle Cell Disease and Thrombosis: A Single Center Experience of Pathophysiology and Potential Risk Factors

**DOI:** 10.3390/hematolrep17050045

**Published:** 2025-09-03

**Authors:** Rafail Tzanninis, Efthymia Vlachaki, Eleftheria Lefkou, Stavroula Tsiara, Stamatia Theodoridou, Athanasios Vyzantiadis, Miltiadis Matsagkas

**Affiliations:** 1 Faculty of Medicine, University of Thessaly, 41334 Larisa, Greece; mmatsagkas@gmail.com; 2 School of Medicine, Aristotle University, 54124 Thessaloniki, Greece; efivlachaki@auth.gr; 3 Transfusion Center, University of Thessaly, 41500 Larisa, Greece; elefkou@uth.gr; 4 Faculty of Medicine, University of Ioannina, 45110 Ioannina, Greece; stsiara@uoi.gr; 5 Hippokration Hospital, 54642 Thessaloniki, Greece; hmesogiaki@ippokratio.gr (S.T.); athanvyzanti@gmail.com (A.V.)

**Keywords:** sickle cell disease, thrombosis, venous thromboembolism

## Abstract

**Background:** Sickle cell disease (SCD) is among the most prevalent inherited hemoglobinopathies and is strongly associated with numerous coagulation abnormalities, hence constituting a severe hypercoagulable state. **Methods**: We conducted a single-center retrospective observational study of patients with SCD who were monitored at Hippokration Hospital of Thessaloniki between 1999 and 2024. Demographic characteristics, hemoglobin (Hb) genotype, medical history, anticoagulant and antiplatelet therapy, dosage of anticoagulant treatment, recurrence of the first episode of venous thromboembolism (VTE) and relevant laboratory values were examined as possible risk factors. **Results:** Among 46 patients, 12 (26.1%) developed thrombosis with the majority (75%) carrying the HbS/β-thal genotype. The prevalence of VTE in this study was 17.4%. Variables significantly associated with an increased risk of thrombosis included age at the time of thrombosis, patient age, use of anticoagulant treatment, anticoagulant dosage, antiplatelet therapy and type of transfusion (*p* < 0.05). On multivariate analysis, anticoagulant treatment and its dosage retained statistical significance (*p* < 0.05). **Conclusions:** These findings reinforce the strong association between SCD and thrombotic events. Despite the availability of a broad therapeutic armamentarium and increasing knowledge of the underlying disease mechanisms, the prevention and management of thrombosis in these patients remains a challenge.

## 1. Introduction

Sickle cell disease (SCD) is a monogenic hemoglobin disorder characterized by the production of the abnormal hemoglobin S [1]. The term encompasses both sickle cell anemia (SCA), which refers to the homozygous state of the sickle cell gene (HbSS) with exclusive HbS production, and the compound heterozygous forms, in which HbS is co-inherited with other hemoglobinopathies [2]. For instance, in HbS/β-thalassemia, one parent contributes the sickle cell gene while the other contributes a β-thalassemia mutation, resulting in double heterozygosity [2].

Venous thromboembolism (VTE), defined as the formation of a blood clot within the venous system, is a well-recognized complication of SCD. Reported prevalence rates in this population reach up to 25%, with multiple risk factors implicated [3,4]. Despite the inherent risk of thrombosis, there are evidence-based guidelines for thromboprophylaxis, while reported utilization of preventive strategies is concerningly low [5].

The pathophysiology of SCD is complex and multifactorial. The central initiating event is HbS polymerization, which induces the transformation of red blood cells into rigid, fragile sickle cells with enhanced adherence to vascular endothelium [6,7,8]. This attachment contributes to vascular occlusion in both small and large vessels [9,10,11,12,13,14]. Concurrently, the fragility of the red blood cells triggers chronic intravascular and extravascular hemolysis resulting in the activation of various cellular populations and the release of signaling molecules, including hemoglobin [1,7,8,15,16]. The fourth component is oxidative stress, which further destabilizes the red cell membranes, thereby accelerating hemolysis and amplifying coagulation activation [8,17]. In addition, disturbances of the coagulation cascade—reduced levels of coagulation factors and natural anticoagulants, fibrin resistance to fibrinolysis and immunothrombosis—play a pivotal role. The secretion of microvesicles and activation of platelets and neutrophils further enhance the hypercoagulable state [7,18].

## 2. Methods

Delving deeper into the interaction of SCD and thrombosis, we conducted a retrospective observational study at the Adult Thalassemia and Sickle Cell Disease Unit of the Hippokration Hospital of Thessaloniki covering the period from 1 January 1999 to 31 November 2024. The primary objectives were to determine the prevalence of thrombosis and VTE among patients with SCD and to evaluate and compare established risk factors.

**Figure 1:**
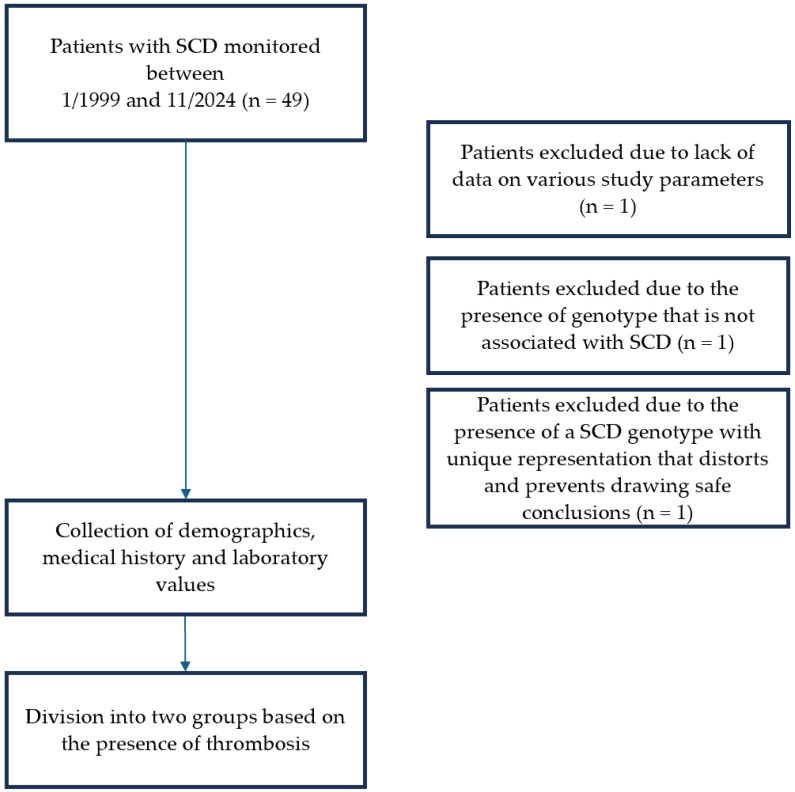
Flowchart of patient inclusion in the study.

Inclusion criteria consisted a confirmed genotype compatible with SCD. Patients with thalassemia syndromes and those with limited follow-up data were excluded due to insufficient laboratory values available for risk factor analysis. Additionally, three patients were excluded after detailed review. One patient lacked adequate clinical and laboratory data as she had recently started monitoring at the Unit. Another patient was excluded following molecular genetic testing that did not confirm an SCD genotype. A third patient carried a complex genotype (compound heterozygosity of sickle cell and β-thalassemia in combination with heterozygous mild α-thalassemia). This genotype was unique within the study. The possible inclusion of this data would significantly alter the statistical outcomes in both univariate and multivariate analyses. For methodological rigor, she was therefore excluded from the final dataset (Figure 1).

Approval for this study was obtained from the hospital’s Ethics Committee, and informed consent for participation was obtained from all subjects involved in the study and was documented through patients’ signatures in their medical records, rather than by a separate consent form. We reviewed all medical files from the Adult Thalassemia and Sickle Cell Disease Unit in detail. Extracted information included duration and etiology of anticoagulant therapy, hemoglobin genotype, history of splenectomy or functional asplenia, demographic characteristics, and comorbid conditions associated with thrombotic risk (Table 1).

Baseline values of hemoglobin, white blood cells, platelets, HbS, and HbF were also recorded prior to the first thrombotic event (Table 2).

## 3. Data Analysis

This study included both quantitative and qualitative variables. Quantitative data are summarized using mean value and measures of dispersion (standard deviation, minimum and maximum value), while qualitative variables are expressed as frequencies and percentages.

Normality of the quantitative variables was assessed using the Shapiro–Wilk and Kolmogorov–Smirnov tests. Given the sample size (~50 patients), both normality tests were applied. For most variables, *p*-values were less than 0.05, indicating rejection of the null hypothesis and lack of a normal distribution. Consequently, non-parametric methods were employed.

Comparisons between two independent groups were performed using the Mann–Whitney U Test and comparisons among more than two groups were conducted using the Kruskal–Wallis test. Associations between categorical variables were examined using crosstabulations with Chi-square tests in order to determine whether the observed differences were random or statistically significant. Finally, linear regression analysis was performed to assess relationships between continuous dependent variables and one or more independent variables.

## 4. Results

### 4.1. Patient Characteristics

A total of 46 patients with SCD monitored at the Adult Thalassemia and Sickle Cell Disease Unit were included in the analysis. Of these, 32 (69.6%) had S/βMA double heterozygosity while 14 (30.4%) had the SCA genotype. The sample was nearly evenly distributed between men and women, ensuring balance in the sex analysis. The mean age at the time of evaluation was 42.9 years (Table 1).

It should be clarified that all percentages and statistical tests referred exclusively to the first episode of thrombosis. Due to the small overall sample size and the even smaller subset of patients with recurrent episodes, statistical modeling in the population with more than one VOE was not feasible, as conclusions would lack robustness. Furthermore, in the context of anticoagulant and antiplatelet treatment, the “before thrombosis” category also included patients who did not experience any thrombotic event during the study period.

### 4.2. Frequency of Thrombotic and Venous Thromboembolic Events

The prevalence of thrombosis, considering only on the first recorded episode, was 26.1% (12/46), while the prevalence of VTE was 17.4% (8/46). The median age at the 1st thrombotic event was 38.1 years (Table 1).

### 4.3. Anatomic Site of First Thrombotic Episode

**Figure 2:**
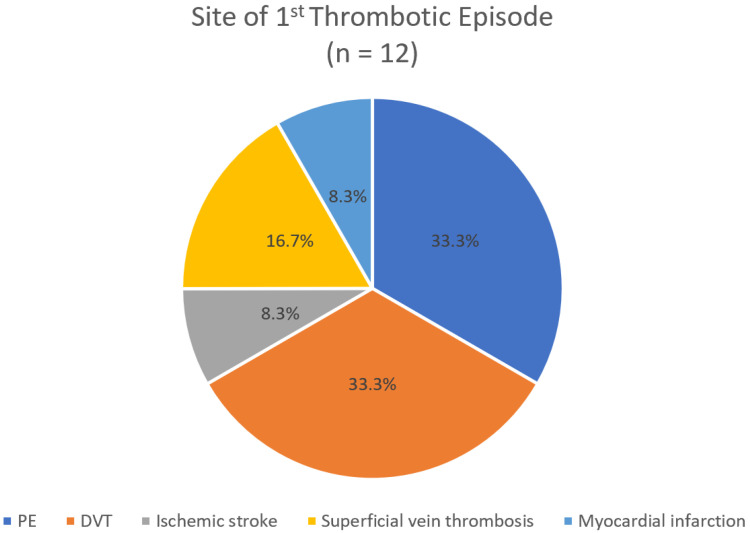
Anatomic classification of the first thrombotic episode.

**Figure 3:**
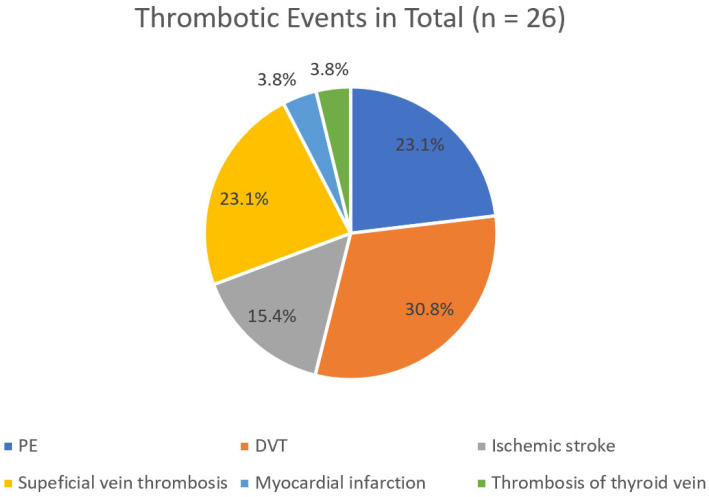
Detailed classification of all VOEs.

It is noteworthy that when analyzing only the first thrombotic episode, PE and DVT were observed at equal frequencies (33.3%) (Figure 2). In contrast, when all 26 thrombotic episodes recorded over the 25-year period of the study were considered, DVT was more common than PE (30.8% > 23.1%) (Figure 3).

### 4.4. Clinical Factors Associated with Thrombosis

Univariate analysis identified several factors significantly associated with thrombosis: patient’s age (*p* = 0.009), age at first VOE (*p* = 0.000), use of antiplatelet treatment (*p* = 0.000), anticoagulant treatment (*p* = 0.000) and anticoagulant dose (*p* = 0.000). In addition, a significant correlation was observed between the type of transfusion and thrombosis (*p* = 0.018) (Table 1).

### 4.5. Laboratory Tests Associated with Thrombosis

In contrast, no laboratory parameters were found to be statistically significant predictors of thrombosis in either univariate or multivariate analyses (Table 2).

Linear regression analysis produced 12 models. Across all models, both the administration of anticoagulants and their dosage were consistently statistically significant predictors of thrombosis. Blood transfusion and transfusion type (regular and exchange) were less consistently significant, with only two models indicating a significant correlation.

### 4.6. Subpopulation with Thrombotic Events

Among the 12 patients who developed thrombosis, there was equal sex representation (six men and six women). The mean age at first episode of thrombosis was 38.1 years, with eight of these events classified as VTE (Table 1).

Nine patients (75%) had S/βMA heterozygosity and three (25%) had SCA. All 12 patients received anticoagulant treatment at therapeutic doses. Ten patients (83.3%) initiated anticoagulants at therapeutic dosage after their first thrombotic event while two (16.7%) were on anticoagulants at the same dose prior to the first event due to underlying conditions (due to a history of AF and tibial artery stenosis). Among the 10 post-event patients, anticoagulant regimens included LMWH for four patients, DOACs for two, VKA for two and fondaparinux for two. The two patients with pre-existing conditions received VKAs. Regarding antiplatelet treatment, five patients (41.7%) initiated treatment after the thrombotic event, three (25%) were already on therapy, and four (33.3%) never received antiplatelet agents. The antiplatelet regimens included COX1 inhibitors for four patients and a P2Y12 inhibitor for one patient. In the subgroup that initiated antiplatelet treatment after the first VOE, two received COX1 inhibitors and one received a P2Y12 inhibitor.

Clinical history revealed that eight patients (66.7%) had conditions associated with thrombogenesis. Four (33.3%) had undergone splenectomy and four (33.3%) had functional asplenia confirmed by abdominal imaging (ultrasound or CT scan). Moreover, seven patients (58.3%) received regular exchange transfusions. All 12 patients were maintained on hydroxyurea throughout the observational period (Table 1).

### 4.7. Subpopulation with More than One Thrombotic Events

Among the 12 patients who experienced thrombosis, five had more than one thrombotic episode. It is worth mentioning that all patients had at least one recurrence at the same anatomical site as the initial event while four (80%) experienced thrombotic events at additional sites. Specifically, one patient (20%) had a second PE, two (40%) had recurrent DVT, one (20%) experienced two superficial thromboses, and two (40%) suffered a second ischemic stroke.

The patient with two episodes of PE also experienced two ischemic strokes. Two patients accounted for the highest number of thrombotic events: one had four events (two episodes of PE and two ischemic strokes) while the other experienced three DVTs (including two recurrences) and three superficial thromboses.

This subgroup shared several common characteristics: (a) all patients had S/βMA double heterozygosity, (b) all received therapeutic anticoagulant treatment after their first thrombotic episode, (c) their baseline Hb was <11 g/dL and (d) all were maintained on hydroxyurea.

The subgroup consisted of three women (60%) and two men (40%). Three patients (60%) received antiplatelet therapy after the first thrombotic episode while two (40%) did not. Two patients (40%) had undergone surgical splenectomy, one (20%) had functional asplenia and two (40%) had neither. Four (80%) were enrolled in an exchange transfusion program. Platelet counts were normal in four patients (80%) and elevated in one (20%), whereas white blood cell counts were elevated in three patients (60%) and normal in two (40%). Four patients (80%) had comorbid conditions potentially contributing to thrombosis. Baseline laboratory values were: PLT: 308 × 10^3^, WBC: 9760, HbS: 47.4%, and HbF: 12%.

The timeline of the subsequent thrombotic episodes varied: four experienced recurrence within 5 years, while one experienced a recurrence within 20 years.

## 5. Discussion

### 5.1. Prevalence of VTE in the Study Population

In this study, the prevalence of thrombosis was 26.1% and the prevalence of VTE was 17.4%, consistent with previously reported ranges in the literature (8–25%). These rates are substantially higher than the 0.1–0.2% annual incidence observed in the general population [5,19].

Considering only the first thrombotic episode, the incidence of PE and DVT was identical (33.3% each). However, when all 26 thrombotic events over the 25-year follow-up were considered, DVT was more frequent than PE (30.8% vs. 23.1%).

### 5.2. Characteristics and Risk Factors of the Study Population

This study reinforces the strong thrombophilic tendency of patients with SCD. The mean age at first thrombotic event was 38.1 years, approximately 10 years older than the 29.9 years reported in the literature [20]. No sex-based differences were observed, and most first thrombotic events occurred in patients with HbS/β-thalassemia heterozygosity (75%), a trend that persisted when all 26 thrombotic events were considered (88.5% vs. 11.5%).

What is interesting is that anticoagulant therapy, and specifically its dosage, was significantly associated with thrombosis in both univariate (both *p* = 0.000) and various multivariate analyses (*p* = 0.000 and *p* = 0.001, respectively). This association likely reflects clinical practice, as patients with thrombosis are more frequently treated with therapeutic anticoagulation. Importantly, seven patients (58.3%) received anticoagulants after their first thrombotic episode and did experience reoccurrence, while four of the five patients with multiple episodes had comorbid conditions (atrial fibrillation, arterial stenosis, venous incompetence of major veins of lower limb, or post-stenotic vascular lesions in the entire lower limb) that could explain recurrent thrombosis. Adding to this fact, five patients receiving anticoagulant treatment for underlying diseases did not experience any VOE.

Recurrences occurred within five years for one patient and within ten years for two patients, which differs from previous reports suggesting higher recurrence rates within five years [21,22].

Although patient age was statistically significant, this observation may be misleading. Examining patients who experienced thrombosis (n = 12), events were more frequent in younger adults (20–39 years, 58.3%) and decreased with age. This finding is consistent with prior studies showing a tropism of thrombosis at younger ages in SCD [23].

While the overall association between transfusion and thrombosis was not significant, the type of transfusion was statistically significant (*p* = 0.018). Among systematically transfused patients, 7/15 who received exchange transfusion experienced thrombosis, compared to 8/15 who did not. Regular transfusion appeared protective, as none of the regularly transfused patients developed thrombosis. These findings suggest a potential link between exchange transfusion and thrombus formation, and between regular transfusion and thrombosis prevention. However, the small sample size may influence these outcomes, and larger studies are required for confirmation. Moreover, these findings may be attributed to the operator-dependent nature of manual exchange transfusions performed at our center, where the procedure carries a slightly increased risk of technical complications, including air embolism, infection, and vascular injury. Finally, factors such as surgical splenectomy and homozygous sickle cell anemia were not significantly associated with thrombosis, in contrast to prior reports, likely due to the limited sample size [23,24,25,26,27].

### 5.3. Characteristics of the Subpopulation That Experienced ≥ Two Thromboses

Five (41.7%) patients experienced more than one episode occurring anywhere from a few months to 17 years apart. The first VTE recurrence occurred within 5 years in one patient and within 10 years in two, which contrasts with the literature. Although the sample size is small and limits robust statistical analysis, noteworthy patterns emerged: the majority of recurrent events occurred in patients with HbS/β-thalassemia heterozygosity, and 10 of the 14 recurrent thrombotic episodes occurred in patients aged ≤45 years.

## 6. Limitations

This study is limited by its retrospective observational design, relying on data extracted from patient medical records. The relatively small sample size may have contributed to the lack of statistically significant correlations for variables previously reported as significant in the literature. The absence of diverse SCD genotypes also limited the generalizability of the findings. In some cases, information regarding the patients’ concomitant diseases was inferred from ongoing therapies rather than directly documented diagnoses. Similarly, the indication for anticoagulant therapy was not always clearly recorded.

For variables such as functional asplenia, imaging was not consistently performed near the time of the thrombotic event; assessments were sometimes based on clinical history, platelet counts, and the most recent ultrasound regardless of timing. Documentation of concomitant PE and DVT was incomplete, and hereditary thrombophilia testing was not reported in 10 of 12 patients with thrombosis.

Furthermore, the temporal relationship between splenectomy and thrombotic events was often unclear. Finally, adherence to prescribed therapies could not be reliably confirmed.

## 7. Conclusions

The distinctive feature of our study is the extended follow-up period and the observation that the highest proportion of patients with thrombosis were individuals with Sβ-thalassemia. This is remarkable, as this population is theoretically considered to have a milder clinical course compared with those with the SS genotype, yet they still developed thrombosis, including multiple events. This finding is particularly important given that, in Greece, the majority of patients have S/βMA double heterozygosity.

Another significant observation, which warrants further investigation, is that patients undergoing exchange transfusion exhibited a higher frequency of thrombosis compared with those managed solely with transfusion therapy. Nevertheless, the limited number of patients in our cohort necessitates studies with larger sample sizes to allow more reliable conclusions. Finally, no bleeding events were recorded.

Sickle cell disease exhibits a strong pathophysiological association with thrombosis, with venous thromboembolism (VTE) as the primary representative. This interaction is complicated by the increased likelihood of recurrence of VTE. Both initial and recurrent thrombotic events tend to occur at a young age and may involve multiple anatomical sites. While anticoagulant therapy reduces the risk of recurrence and new thrombotic events, it does not eliminate thrombosis or nullify the underlying hypercoagulable state. Despite advances in understanding the disease’s pathogenesis and the improvements of anticoagulant treatment, the innate complexity of SCD continues to pose challenges in preventing thrombotic complications, which can range from functional impairment to mortality.

## Figures and Tables

**Table 1:** Demographic and clinical data of study population.

Patients’ Characteristics	Detailed Information Regarding Patients’ Characteristics	Patients in Total (N = 46)	Patients with VOE (n = 12)	Patients with No VOE (n = 34)	*p*-Value
Age of patients (Years)		42.9	47	41	0.009
Age of thrombosis (Years)			38.1	-	0.000
Sex	Male	24 (52.2%)	6 (50%)	18 (52.9%)	0.861
	Female	22 (47.8%)	6 (50%)	16 (47.1%)	0.861
Genotype	SCA	14 (30.4%)	3 (25%)	11 (32.4%)	0.63
	Double heterozygosity S/βMA	32 (69.6%)	9 (75%)	23 (67.6%)	0.64
Site of thrombosis		12 (26.1%)	-	0%	0.000
	PE	4 (8.7%)	4 (33.3%)	-	-
	DVT	4 (8.7%)	4 (33.3%)	-	-
	Other	4 (8.7%)	4 (33.3%)	-	-
Recurrent episode of DVT (1)		1 (2.2%)	1 (8.3%)	-	-
Recurrent episodes of DVT (>1)		2 (4.3%)	2 (16.7%)	-	-
≥2 thrombotic episodes		5 (10.9%)	5 (41.7%)	-	-
Anticoagulant treatment		17 (37%)	12 (100%)	5 (14.7%)	0.000
	Before 1st VOE	7 (17.4%)	2 (16.7%)	-	-
	After 1st VOE	10 (21.7%)	10 (83.3%)	-	-
	No anticoagulant treatment	29 (63%)	0 (0%)	29 (85.3%)	-
Antiplatelet treatment		25 (54.3%)	8 (66.7%)	17 (50%)	0.000
	Before 1st VOE	20 (43.5%)	3 (25%)	-	-
	After 1st VOE	5 (10.9%)	5 (41.7%)	-	-
	No antiplatelet treatment	21 (47.5)	4 (33.3%)	17 (50%)	0.000
Anticoagualants’ dosage		17 (37%)	12 (100%)	5 (14.7%)	0.000
	Prophylactic	3 (6.5%)	0 (0%)	3 (8.8%)	-
	Therapeutic	14 (30.4%)	12 (100%)	2 (5.9%)	-
Medical history could be the cause thrombosis	Yes	28 (60.9%)	8 (66.7%)	20 (58.8%)	0.63
	No	18 (39.1%)	4 (33.3%)	14 (41.2%)	0.63
Surgical splenectomy		20 (43.5%)	4 (33.3%)	16 (47.1%)	0.405
Fuctional asplenia		15 (32.6%)	4 (33.3%)	11 (32.4%)	0.95
Transfusion		23 (50%)	7 (58.3%)	16 (47.1%)	0.501
Type of transfusion		23 (50%)	7 (58.3%)	16 (47.1%)	0.018
	Exchange transfusion	15 (32.6%)	7 (58.3%)	8 (23.5%)	-
	Packed RBC transfusion	8 (17.4%)	0 (0%)	8 (23.5%)	-
	No transfusion	23 (50%)	5 (41.7%)	18 (52.9%)	-
Use of hydroxycarbamide		46 (100%)	12 (100%)	34 (100%)	-

DVT: deep vein thrombosis, VOE: vaso-occlusive event, SCA: sickle cell anemia.

**Table 2:** Laboratory values of study group.

Laboratory Tests	Hb Value	Patients in Total (N = 46)	Patients with VOE (n = 12)	Patients with No VOE (n = 34)	*p*-Value
Hb (g/dL) [11.5–14.5 × 10^6^ g/L]	<11	39 (84.8%)	10 (83.3%)	29 (85.3%)	0.872
	>11	7 (15.2%)	2 (16.7%)	5 (14.7%)	0.872
WBC (10^3^/μL) [4.5–10.5 × 10^3^/μL]		9.1 (5.3)	9.1 (3.4)	9.1 (5.9)	0.910
Plt (10^3^/μL) [150–400 × 10^3^/μL]		384.6 (159.2)	397.1 (168.6)	349.2 (128.7)	0.474
HbS (%) [0%]		60.4 (15)	55.9 (16.4)	62 (14.5)	0.706
HbF (%) [<2%]		13.7 (8.9)	13.8 (6.9)	13.6 (9.7)	0.239

WBC: white blood cells, Plt: platelets, HbS: hemoglobin S, HbF: hemoglobin F. For the measurement of the patients’ HbS and HbF, high-performance liquid chromatography was performed.

## Data Availability

Data supporting the findings are subject to privacy and ethical restrictions and, therefore, cannot be made publicly available.

## Abbreviations

The following abbreviations are used in this manuscript:

ACS	Acute Chest Syndrome
AF	Arial Fibrillation
DOAC	Direct Oral Anticoagulant
DVT	Deep Venous Thrombosis
Hb	Hemoglobin
LMWH	Low Molecular Weight Heparin
PE	Pulmonary Embolism
RBC	Red Blood Cell
SCA	Sickle Cell Anemia
SCD	Sickle Cell Disease
VKA	Vitamin K Antagonist
VOE	Vaso-occlusive Event
VTE	Venous Thromboembolism
